# Conventional myelosuppressive chemotherapy for non-haematological malignancy disrupts the intestinal microbiome

**DOI:** 10.1186/s12885-021-08296-4

**Published:** 2021-05-22

**Authors:** Lito E. Papanicolas, Sarah K. Sims, Steven L. Taylor, Sophie J. Miller, Christos S. Karapetis, Steve L. Wesselingh, David L. Gordon, Geraint B. Rogers

**Affiliations:** 1 South Australian Health and Medical Research Institute, Adelaide, South Australia Australia; 2 South Australian Health and Medical Research Institute Microbiome Research Laboratory, College of Medicine and Public Health, Flinders University, Bedford Park, South Australia 5042 Australia; 3 Flinders Centre for Innovation in Cancer, Flinders University, Bedford Park, South Australia Australia; 4 Department of Medical Oncology, Flinders Medical Centre, Bedford Park, South Australia Australia; 5 Microbiology and Infectious Diseases, Flinders Medical Centre, Bedford Park, South Australia Australia

**Keywords:** Chemotherapy, Cancer, Microbiome

## Abstract

**Background:**

The gut microbiota influences many aspects of host physiology, including immune regulation, and is predictive of outcomes in cancer patients. However, whether conventional myelosuppressive chemotherapy affects the gut microbiota in humans with non-haematological malignancy, independent of antibiotic exposure, is unknown.

**Methods:**

Faecal samples from 19 participants with non-haematological malignancy, who were receiving conventional chemotherapy regimens but not antibiotics, were examined prior to chemotherapy, 7–12 days after chemotherapy, and at the end of the first cycle of treatment. Gut microbiota diversity and composition was determined by 16S rRNA gene amplicon sequencing.

**Results:**

Compared to pre-chemotherapy samples, samples collected 7–12 days following chemotherapy exhibited increased richness (mean 120 observed species ± SD 38 vs 134 ± 40; *p* = 0.007) and diversity (Shannon diversity: mean 6.4 ± 0.43 vs 6.6 ± 0.41; *p* = 0.02). Composition was significantly altered, with a significant decrease in the relative abundance of gram-positive bacteria in the phylum Firmicutes (pre-chemotherapy median relative abundance [IQR] 0.78 [0.11] vs 0.75 [0.11]; *p* = 0.003), and an increase in the relative abundance of gram-negative bacteria (Bacteroidetes: median [IQR] 0.16 [0.13] vs 0.21 [0.13]; *p* = 0.01 and Proteobacteria: 0.015 [0.018] vs 0.03 [0.03]; *p* = 0.02). Differences in microbiota characteristics from baseline were no longer significant at the end of the chemotherapy cycle.

**Conclusions:**

Conventional chemotherapy results in significant changes in gut microbiota characteristics during the period of predicted myelosuppression post-chemotherapy. Further study is indicated to link microbiome changes during chemotherapy to clinical outcomes.

**Supplementary Information:**

The online version contains supplementary material available at 10.1186/s12885-021-08296-4.

## Background

Gut microbiome characteristics are predictive of cancer treatment outcomes, including response to myelosuppressive chemotherapies and immunotherapies [1–3], and severe adverse events including sepsis of gut origin [4]. However, surprisingly little is known about the direct impacts of chemotherapeutic agents on the gut microbiome, as a potential mediator of treatment outcomes.

Chemotherapeutic agents have been known to inhibit bacterial growth for many decades. For instance, the inhibitory effects of cisplatin on *E. coli* preceded identification of its anti-tumour effects [5]. The in-vitro effects of cancer chemotherapeutics on a wide range of commensal bacteria have recently been demonstrated by Maier et al. [6]. However, few studies have attempted to assess the effect of chemotherapy on intestinal microbiology in humans [7–9]. Moreover, the majority of patients in these studies were being treated for haematological malignancy and also received prophylactic antibiotics immediately preceding, or during, the study period. These studies were therefore unable to attribute microbiological effects to chemotherapy alone.

Our aim was to determine whether conventional myelosuppressive chemotherapy alters intestinal microbiota characteristics in patients with solid organ malignancy, in the absence of antibiotics or other exposures that may independently disrupt intestinal microbiology.

## Methods

Ethics approval for the study was received from the Southern Adelaide Local Health Network Human Research Ethics Committee (HREC/17/SAC/44). This prospective pilot observational cohort study recruited participants between February 2018 and July 2019 at a single primary referral centre. Chemotherapy-naïve patients, commencing the first cycle of conventional myelosuppressive chemotherapy for a non-haematological malignancy, were invited to participate. Participants who received antibiotics within 4 weeks of chemotherapy (a period associated with gut microbiota disruption following antibiotic exposure [10]), were excluded. Participants with other potentially confounding exposures, including prior chemotherapy, immunotherapy, malignancy involving the gastro-intestinal lumen, inflammatory bowel disease or probiotic use were excluded. Faecal samples were self-collected using nucleic acid preservation tubes (Norgen Biotek Corp, Thorold, ON, Canada) prior to commencement of chemotherapy (pre-chemo: median 1 day preceding chemotherapy; IQR 2), 7–12 days after chemotherapy (median 9 days; IQR 2), and at the end of the first chemotherapy cycle (median 21 days post chemotherapy; IQR 8.5).

### DNA extraction

Stool was weighed, and DNA extracted using the DNeasy PowerSoil HTP 96 DNA Isolation kit (Qiagen, Chadstone VIC, Australia; Cat No. 12888–100). The following modification to the manufacturer’s instructions were employed: samples and solution C1 were added into bead tubes and heated for 10 min at 65 °C, prior to two cycles of bead beating at 6.5 m/s for 1 min using a FastPrep-24 bead beater (MP Biomedicals, Santa Ana, CA, USA). Quant-IT dsDNA Assay kit (Life Technologies, Carlsbad, CA, USA) was used to quantify DNA concentration after extraction. Extracted DNA was stored at − 20 °C prior to further analysis.

### Total bacterial load and *E. coli* quantitation

Quantitation of total bacteria was performed using previously described universal primers targeting the bacterial 16S rRNA gene [11] and PowerUp SYBR Green qPCR Master Mix reagents (ThermoFisher, Cat No., Foster City, CA, USA). *E. coli* DNA was amplified using a previously described probe-based assay [12] using KAPPA PROBE FAST ROX Low Master Mix reagents (Kapa Biosystems, Cape Town, South Africa). Real-time PCR quantitation was performed using the QuantStudio 6 Real-Time PCR system (Applied Biosystems, Foster City, CA, USA). Total bacteria and *E. coli* (per gram of stool) were quantified by comparing sample Ct to a standard curve using DNA extracted from a known quantity of *E. coli* (ATCC strain 36,218).

### 16S rRNA gene amplicon sequencing

Faecal microbiome characteristics were determined by sequencing the V4 hypervariable region of the 16S rRNA gene bacterial gene using next-generation amplicon sequencing (Illumina MiSeq) as described previously [13]. Raw sequences have been uploaded to the National Center for Biotechnology Information (https://www.ncbi.nlm.nih.gov/) under BioProject ID PRJNA650259.

Demultiplexed paired-end reads were denoised and quality filtered using DADA2 [14] and amplicon sequence variants (ASVs) were assigned a taxonomy by alignment to the SILVA database (v132) at 97% sequence similarity using Quantitative Insights in to Microbial Ecology (QIIME) software (v2.2019.4) [15]. Reads aligning with contaminants, including mitochondria, eukaryota, chloroplast and cyanobacteria were removed. Median read depth after filtering was 11,510 (IQR 7078). The taxa relative abundances were calculated at the phyla and genus levels on unrarefied data. Metrics for determining α-diversity (observed species, Shannon and Faith’s PD) and Bray-Curtis dissimilarity distances (where 0 indicates sample composition is identical and 1 indicates there are no shared species) were computed using QIIME v2.2019.4, using the “qiime diversity core-metrics-phylogenetic” command, rarefied to 4846 reads.

To control for natural temporal variability in intestinal microbiome characteristics that occurs with repeated sampling, Bray-Curtis dissimilarity distances were also determined on faecal samples collected at matching time intervals from six healthy participants not exposed to chemotherapy. These samples were processed in an identical manner to samples from the chemotherapy exposed cohort. The screening and recruitment for these healthy faecal donors have been published previously [16]. Apart from sampling interval, these participants were not otherwise matched to the participants receiving chemotherapy. Other microbiota characteristics including diversity and composition of samples from healthy participants were therefore not compared to that of the chemotherapy cohort. For this analysis, each participant’s own pre-chemotherapy sample served as the baseline comparator sample.

### Statistical analysis

Significance of pre and post-chemotherapy inter-sample variance (β-diversity) was determined using Bray-Curtis similarity on square root transformed taxa relative abundance using PRIMER software version 7 (PRIMER-E, Plymouth, UK). Other statistical analyses were performed using GraphPad Prism 7.03 software. Participant-specific longitudinal changes were assessed by paired t-tests for parametric data, and the Wilcoxon matched-pairs signed rank test for non-parametric data. For comparisons between unpaired samples, unpaired t-test or were used for parametric data, and the Mann-Whitney U test for non-parametric data. Significance values were adjusted using the Benjamini-Hochberg correction for multiple testing, and a threshold *p* < 0.05 employed. Results were visualised using GraphPad Prism 7.03 software or R.

## Results

Twenty-four patients were enrolled. Five were unable to produce pre-chemotherapy samples and were excluded from analysis. One patient died following collection of the first post-chemotherapy sample and the final specimen was therefore not collected. The cohort consisted of 12 females and 7 males with ages ranging from 48 to 82 years old (mean 68 ± SD 8.7). The number of patients with each type of malignancy and types of chemotherapy used, are presented in Table 1.

**Table 1:** Number of patients in the cohort with cohort with each malignancy type and chemotherapy regimen used

	No.
**Malignancy type**	
Breast	4
Non-small-cell lung cancer	4
Pancreatic	2
Bladder	1
Cholangiocarcinoma	1
Endometrial	1
Osteosarcoma	1
Mesothelioma	1
Small-cell lung cancer	1
Urothelial	1
Unknown primary	1
**Chemotherapy regimen**	
Platinum agent + Gemcitabine	6
Doxorubicin + Cyclophosphamide + Paclitaxel	4
Platinum agent + Etoposide	2
Cisplatin + Doxorubicin	1
Capecitabine	1
Carboplatin + Paclitaxel	1
Cisplatin + Pemetrexed	1
Oxaliplatin + Irinotecan +5FU	1
Abraxane	1

### Impact of chemotherapy on microbiota characteristics

**Fig. 1:**
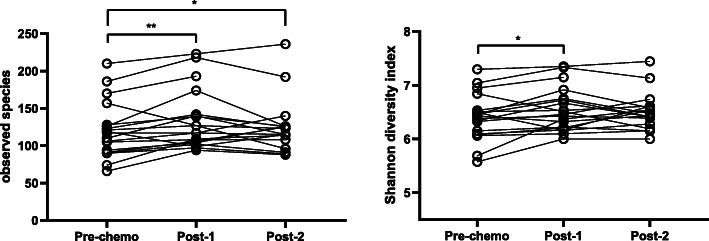
Paired sample α-diversity changes during chemotherapy. **a** Observed species as a measure of bacterial richness and **b** Shannon diversity index as a measure of bacterial diversity. Pre-chemo: baseline samples (prior to chemotherapy), Post-1: 7–12 days post start of chemotherapy, Post-2 at the end of one chemotherapy cycle (median 21 days after chemotherapy). * = *p* < 0.05; ** = *p* < 0.01, performed Wilcoxon matched pairs signed rank test of 19 paired subject samples

The absolute number of bacteria per unit volume in faecal samples did not change with chemotherapy (pre-chemotherapy median 1.14 × 10^9^ bacterial cells/g stool [IQR 2.3 × 10^9^] vs median 1.6 × 10^9^ cells/g stool [IQR 1.6 × 10^9^] 7–12 days post-chemotherapy; *p* = 0.76). However, there was a significant increase in within-sample microbial diversity (α-diversity) following chemotherapy (Fig. 1). Observed bacterial richness (mean 120 ± SD 38 observed species vs 134 ± 40; *p* = 0.007) and Shannon diversity (mean 6.4 ± 0.43 vs 6.6 ± 0.41; *p* = 0.02), were significantly higher 7–12 days after chemotherapy. Increased bacterial richness persisted to the end of the chemotherapy cycle (mean 125 observed species ± SD 36 *p* = 0.02).

### Impact of chemotherapy on microbiome composition

**Fig. 2:**
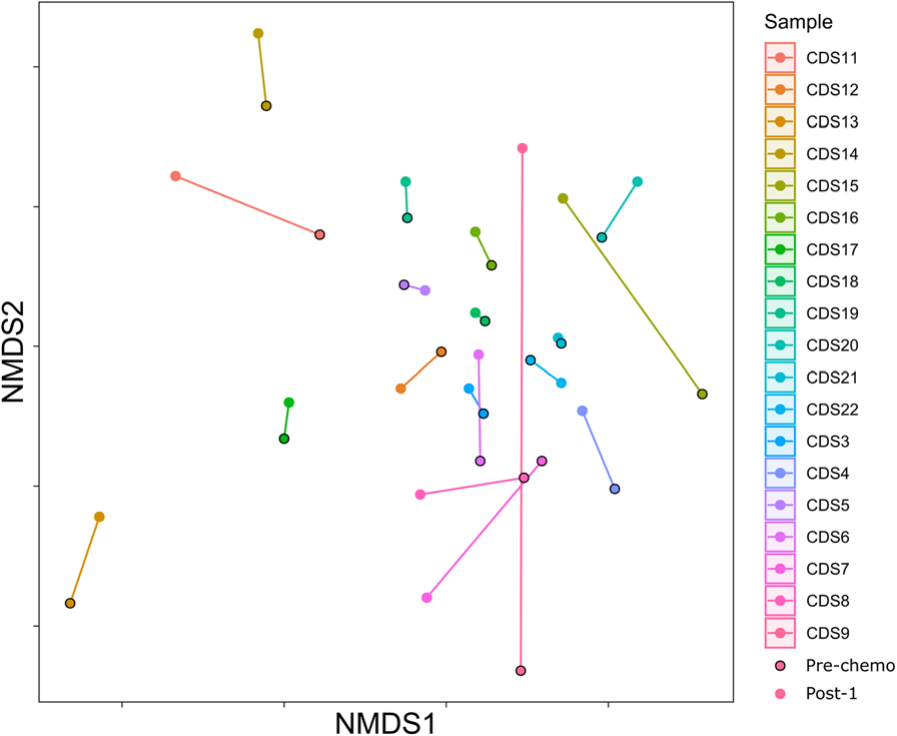
Non-metric multi-dimensional scaling (nMDS) plot showing paired-sample changes to microbiota composition following 7–12 days of chemotherapy (Post-1). Each colour represents an individual participant, with the pre-chemo sample (outline, lighter shade) linked to the post-chemotherapy sample (no outline, solid shade) by a line. Samples are shown to cluster by participant rather than by sampling time point, with no significant difference between the pre-chemo and post-1 groups PERMANOVA; *p* = 0.99

**Fig. 3:**
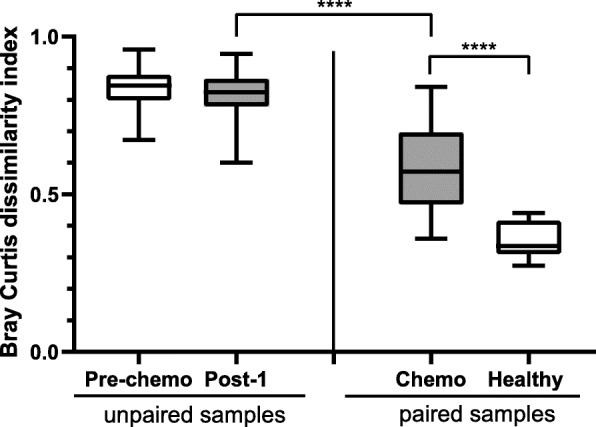
The box plot figure depicts the median, IQR and range of the degree of similarity of the microbiomes in groups of samples using the Bray Curtis dissimilarity index where 0 indicates sample composition is identical and 1 indicates there are no shared species. The degree of similarly in samples from different participants in the cohort before chemotherapy (pre-chemo, unpaired) and 7–12 days following chemotherapy (post-1, unpaired) is depicted on the left. This shows that individual participant’s microbiomes were very different from each other before chemotherapy and remained very different (with no significant change in the degree of dissimilarity) following chemotherapy. On the right the degree of similarity between paired samples from the same participants before and 7–12 days after chemotherapy (chemo, paired) or healthy participants (healthy, paired) at matching sampling intervals are depicted. This shows that participant microbiomes were more similar to their own matched sample than to unrelated samples, but that the degree of difference in within-participant microbiomes before and after chemotherapy was significantly greater than that of paired samples from healthy participants. Significant comparisons are indicated by stars (**** = *p* < 0.0001; one-way ANOVA)

There were no significant differences in the distribution or dispersion of bacterial communities (β-diversity) before chemotherapy compared to after chemotherapy (PERMANOVA *p* = 0.99 and PERMDISP *p* = 0.90 for comparisons with baseline vs 7–12 days post chemotherapy) (Supplemental Figure 1), with samples clustering by participant, rather than time point (Fig. 2). Between-participant differences in microbiota composition were not significantly different before and after chemotherapy (mean Bray-Curtis dissimilarity distance 0.84 ± SD 0.06 pre-chemo vs mean 0.81 ± SD 0.06 post-1; Fig. 3, *p* = 0.07).

`````````````````````````

The change in microbiota composition from pre- to 7–12 days post-chemotherapy, within the same participant, was significantly less than the difference between participants at either sampling timepoint (mean Bray-Curtis dissimilarity distance 0.58 ± SD 0.14 vs 0.81 ± SD 0.06; Fig. 3, *p* < 0.0001). However, compared to microbiota composition from healthy participants who did not receive chemotherapy, where samples were collected at similar timepoints, there was a significantly greater change in microbiota composition in those receiving chemotherapy (mean Bray-Curtis dissimilarity distance 0.35 ± SD 0.14 vs 0.58 ± SD 0.14; Fig. 3, *p* < 0.0001).

### Impact of chemotherapy on specific bacterial taxa

**Fig. 4:**
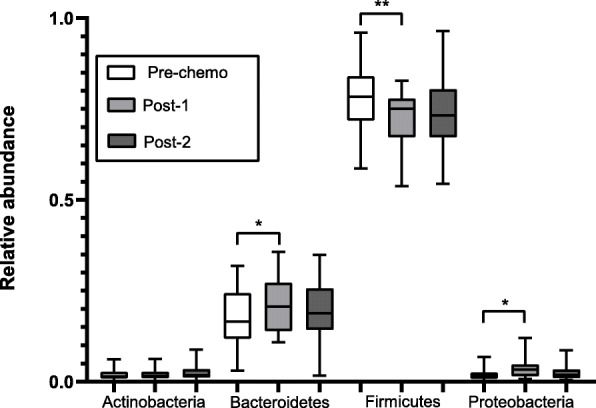
Effect of chemotherapy on microbiome composition: Phyla relative abundance. The relative abundance of the four most abundant bacterial phyla (Firmicutes, Bacteroidetes, Proteobacteria and Actinobacteria) representing 97% of bacteria in the samples, were analysed. Pre-chemotherapy faecal microbiome composition (Pre-chemo) was compared to 7–12 days post-chemotherapy faecal microbiome composition (Post-1) and to faecal microbiome composition at the end of a chemotherapy cycle (median 21 days post-chemotherapy, Post-2) in 19 participants. Box and whiskers depict median ± interquartile range with bars representing minimum and maximum values. All significant comparisons are indicated by stars (* = *p* < 0.05; ** = *p* < 0.01; *** = *p* < 0.001; Wilcoxon matched-pairs signed rank test)

The microbiota of all chemotherapy participant samples consisted of 11 bacterial phyla (Supplemental Figure 2). We analysed changes in the four most abundant phyla (Firmicutes, Bacteroidetes, Proteobacteria and Actinobacteria), which together represent the majority (median 99.3%, IQR 2.0%) of bacteria in the samples. Of these, the relative abundance of the gram-positive Firmicutes phylum was reduced 7–12 days post-chemotherapy (pre-chemotherapy median relative abundance 0.78, IQR 0.11 vs 0.75, IQR 0.11; *p* = 0.003), while the relative abundance of gram-negative phyla was increased (Bacteroidetes: median 0.16, IQR 0.13 vs 0.21, IQR 0.13; *p* = 0.01 and Proteobacteria: 0.015, IQR 0.018 vs 0.03, IQR 0.03; *p* = 0.02). Levels of these phyla were no longer significantly different to baseline levels at the end of the chemotherapy cycle (Fig. 4).

At the genus level, 259 individual taxa were identified, of which 95 were present in ≥20% of samples and analysed further (Supplemental Figure 3). Of these, the relative abundance of three genera changed significantly from baseline to 7–12 days post-treatment (*p* < 0.05, uncorrected). Two members of the Firmicutes phylum decreased in relative abundance: Ruminococcaceae UCG-014 (median [IQR], 0.002 [0.02] vs 0 [0.004]; *p* = 0.006) and Clostridia D_3_Clostridiales (unnamed genus) (0.021 [0.07] vs 0.011 [0.05]; *p* = 0.025) and the genus Bacteroides of the Bacteriodetes phylum increased in relative abundance (0.123 [0.11] vs 0.153 [0.11]; *p* = 0.03). However, these differences were no longer significant after correction for multiple testing.

**Fig. 5:**
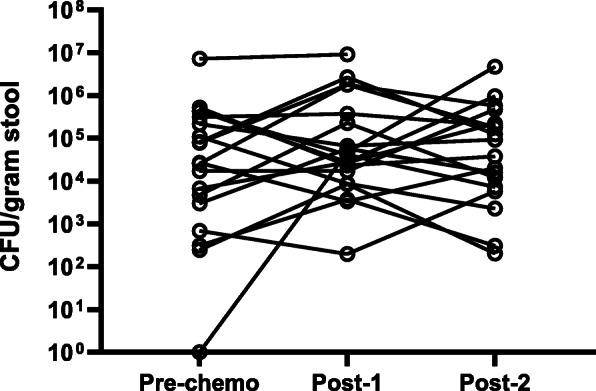
Absolute abundance of *E. coli* bacteria determined using *E. coli* specific qPCR. Total *E. coli* equivalent colony forming per gram of stool (CFU/gram stool) of each participant’s stool sample was assessed at three time points before chemotherapy (Pre-chemo), 7–10 days post chemotherapy (Post-1) and at the end of one chemotherapy cycle (Post-2). Dots represent individual values of 19 paired subject samples. The increase in *E. coli* absolute abundance following chemotherapy was not significant (*p* = 0.18; paired t-test)

The absolute abundance of *E. coli* was selected to be quantified as this is the most common intestinal human commensal and pathogen represented in the phylum Proteobacteria*. E. coli* absolute abundance did not change significantly following chemotherapy at either sampling time point (log10 CFU/μL mean ± SD: 4.3 ± 1.6 pre-chemotherapy vs 4.8 ± 1.6 7–12 days post-chemotherapy; *p* = 0.18, and vs 4.6 ± 1.2 at the end of the chemotherapy cycle; *p* = 0.28, Fig. 5).

## Discussion

The importance of assessing chemotherapy-associated changes in the absence of antibiotic exposure is highlighted by the substantial differences between our findings and those of previous studies in which patients received both chemotherapy and antibiotics [7, 9]. We did not observe a fall in the absolute number of bacteria in the faecal samples following chemotherapy, as has been reported previously, or a decrease in α-diversity [8, 17]. Reduced α-diversity has been independently linked with antibiotic use in patients with haematological malignancy [18, 19] and is associated with worse clinical outcomes, including increased risk of infection [18] and increased overall mortality [17]. The increase in gut microbial diversity following chemotherapy in the absence of antibiotic exposure, reported here, is therefore reassuring.

In the absence of a driver of microbiome disruption, such as exposure to antibiotics, the composition of an individual’s gut microbiome typically remains relatively stable over time [20, 21]. However, paired analysis of microbiome composition before and after chemotherapy showed that microbiome composition variability was greater than expected, as there was far less temporal change occurring in healthy participants who did not receive chemotherapy over the same time interval. This suggests that chemotherapy, rather than natural temporal variability of samples, drives the changes to microbiome composition.

We identified phyla-specific effects of chemotherapy. There was a decrease in the relative abundance of gram-positive bacteria in the phylum Firmicutes 7–12 days post-treatment, and an increase in the relative abundance of gram-negative bacteria in the Bacteroidetes and Proteobacteria phyla. A similar relative decrease in Firmicutes and relative increase in Proteobacteria following chemotherapy for haematopoietic stem cell transplant has been previously been reported by Montassier et al. [8], while decreases in the relative abundance of Clostridium cluster XIVa within the Firmicutes phylum was reported by Zwielehner et al. [9].

Decreased abundance of Firmicutes following chemotherapy was also observed in Viaud et al’s landmark study [1], where it was demonstrated that cyclophosphamide treatment in mice resulted in disruption of the intestinal barrier, a decline in Firmicutes abundance, translocation of Firmicutes bacteria into lymphoid organs, and the activation of T-cell immune responses with antitumor effects [1]. The reported ability to reverse these antitumor effects using antibiotics highlights the importance of commensal microbiota in triggering host anti-tumour immune responses. In this study, a decline in Firmicutes was also observed within days of chemotherapy, mirroring the findings of Viaud et al. Therefore, it is possible that the translocation of Firmicutes bacteria from the gut also play a part in driving antitumor responses in humans receiving conventional chemotherapy.

Anaerobic commensal bacteria produce a range of short-chain fatty acids (SCFA) including acetate, butyrate and propionate. SCFA have a diverse range of beneficial effects on host physiology, including maintenance of gut barrier integrity, promotion of host anti-tumour responses, and suppression of pathogen overgrowth [22]. A range of bacteria in the Firmicutes phylum are the responsible for the biosynthesis of the SCFA butyrate, the metabolite most closely linked with immune regulation and host anti-tumour responses [22]. Depletion of these species has been linked to increased mortality in patients with haematological malignancy [23]. In this study, although a general decline in Firmicutes abundance was observed, we did not find a specific decline in butyrate-producing species. Therefore, it is unlikely the changes we observed led to a change in SCFA levels in the gut. However, if this trend persisted over several cycles of chemotherapy, there is the possibility that the SCFA balance in the gut could change to favour propionate (produced by Bacteriodetes bacteria) at the expense of lower butyrate production by the Firmicutes.

The changes we observe in our study are likely to inform the risk of infection occurring during chemotherapy when innate immune defences are compromised and damage to the intestinal epithelium facilitates bacterial translocation [24]. Proteobacteria, particularly pathogenic members of the Enterobacteriaceae family, such as *E. coli,* are common causes of gut-derived infection. Increased relative abundance of Proteobacteria, a change observed in this study, has previously been linked to adverse infectious outcomes in patients with haematological cancers [18, 25].

In patients with haematological malignancy, a baseline composition of > 5% Enterobacteriaceae has been linked to sepsis while < 10% Lachnospiraceae (from the Firmicutes) is associated with overall mortality [23]. No participants in this cohort had a composition of < 10% Lachnospiraceae, however one participant’s faecal microbiome consisted of > 5% Enterobacteriaceae (6% pre-chemotherapy and 10% 7–12 days following chemotherapy). This was the only participant who developed sepsis and died during the study period. This supports the hypothesis that markers of increased pathogen prevalence in the gut microbiota could serve as markers to predict infectious outcomes in other types of cancer patients.

The changes observed in this study were most pronounced 7–12 days after chemotherapy, a timepoint at which patients are neutropaenic and at increased risk of systemic infection. Indeed, it may be that changes in host immune function occurring during myelosuppression are driving changes in microbiota. At the same time, translocation of bacteria through the gut epithelium are likely to be important mediators of therapy-associated host anti-tumour responses [1]. Therefore, gut microbial composition at this time point is likely to be particularly important in determining risk of infection, but may also be important in mediating chemotherapy efficacy.

A limitation of this study is the small sample size and the inclusion of many types of malignancy and therefore the inability to relate microbiome changes observed to clinical outcomes. To move forward, the relationship between chemotherapy-induced gut microbiome changes and the use of specific chemotherapeutic regimens resulting in different degrees of myelosuppression must now be determined through assessment of larger patient cohorts. These analyses should be conducted over multiple chemotherapy cycles with the aim of linking the changes occurring to microbiome composition with outcomes including response to chemotherapy and the risk of developing adverse effects such as colitis and sepsis.

Unlike many other variables that influence health outcomes, the microbiome is modifiable. For example, pre-biotics can be used to enrich the growth of beneficial bacteria, targeted antimicrobials selectively eradicate pathogenic bacteria with minimal effect on other commensals, and faecal transplants, are able to reconstitute entire microbiomes. By understanding the links between chemotherapy, longitudinal changes occurring in the gut microbiome and clinical outcomes it may be possible to more accurately predict outcomes of chemotherapy and develop microbiome-targeted interventions to reduce infection risk and augment treatment efficacy.

## Conclusions

Following chemotherapy, the gut microbiome is disrupted far more than expected in the sampling time interval tested. Although each individual’s microbiota changed in different ways, small but significant decreases in the relative abundance of gram-positive bacteria in the phylum Firmicutes and corresponding increases in the relative abundance of in bacteria in the gram-negative bacteria in the phyla Bacteriodetes and Proteobacteria were consistently observed. These changes are likely to increase the risk of infectious adverse outcomes but may also represent beneficial changes linked to host anti-tumour immune responses.

## Supplementary Information

**Additional file 1: Supplementary Figure 1.** Non-metric multi-dimensional scaling (nMDS) plot showing between group comparisons of faecal microbiome distribution and dispersal. nMDS plots depicted from Bray-Curtis resemblance of square root transformed, genus-level, relative abundance data. Shaded ovals represent 80% confidence interval. There is no difference between the pre-chemotherapy and post-chemotherapy (post-1) faecal microbiome distribution (PERMANOVA; *p*=0.99) or dispersion (PERMDISP; *p*=0.90). **Supplementary Figure 2.** Taxa bar plot showing the 11 phyla detected. Grouped by time point with Pre=pre-chemotherapy, Post1=7-12 days following chemotherapy, and Post2= at the end of a chemotherapy cycle (median 21 days post-chemotherapy). Showing predominance of Bacteroidetes and Firmicutes. **Supplementary Figure 3.** Taxa bar plot showing the 95 genera detected in ≥20% of samples. Grouped by time point with Pre = pre-chemotherapy, Post1 = 7–12 days following chemotherapy, and Post2 = at the end of a chemotherapy cycle (median 21 days post-chemotherapy).

## Data Availability

All data associated with this study are available from the authors on reasonable request. Raw sequences and associated de-identified participant meta-data have been uploaded to (https://www.ncbi.nlm.nih.gov/) under BioProject ID PRJNA650259.

## Abbreviations

IQR: Interquartile range
SD: Standard deviation
PCR: Polymerase chain reaction
CFU: Colony forming unit
