# Supplementary material for: Conventional myelosuppressive chemotherapy for non-haematological malignancy disrupts the intestinal microbiome
Source: BMC Cancer. 2021 May 22;21:591. doi: 10.1186/s12885-021-08296-4 (PMC8141218; doi:10.1186/s12885-021-08296-4)
Supplement: Supplementary file 1 — Additional file 1: Supplementary Figure 1. Non-metric multi-dimensional scaling (nMDS) plot showing between group comparisons of faecal microbiome distribution and dispersal. nMDS plots depicted from Bray-Curtis resemblance of square root transformed, genus-level, relative abundance data. Shaded ovals represent 80% confidence interval. There is no difference between the pre-chemotherapy and post-chemotherapy (post-1) faecal microbiome distribution (PERMANOVA; p=0.99) or dispersion (PERMDISP; p=0.90). Supplementary Figure 2. Taxa bar plot showing the 11 phyla detected. Grouped by time point with Pre=pre-chemotherapy, Post1=7-12 days following chemotherapy, and Post2= at the end of a chemotherapy cycle (median 21 days post-chemotherapy). Showing predominance of Bacteroidetes and Firmicutes. Supplementary Figure 3. Taxa bar plot showing the 95 genera detected in ≥20% of samples. Grouped by time point with Pre = pre-chemotherapy, Post1 = 7–12 days following chemotherapy, and Post2 = at the end of a chemotherapy cycle (median 21 days post-chemotherapy). [file 12885_2021_8296_MOESM1_ESM.docx]

**Conventional myelosuppressive chemotherapy for non-haematological malignancy disrupts the intestinal microbiome**

**SUPPLEMENTARY DATA**


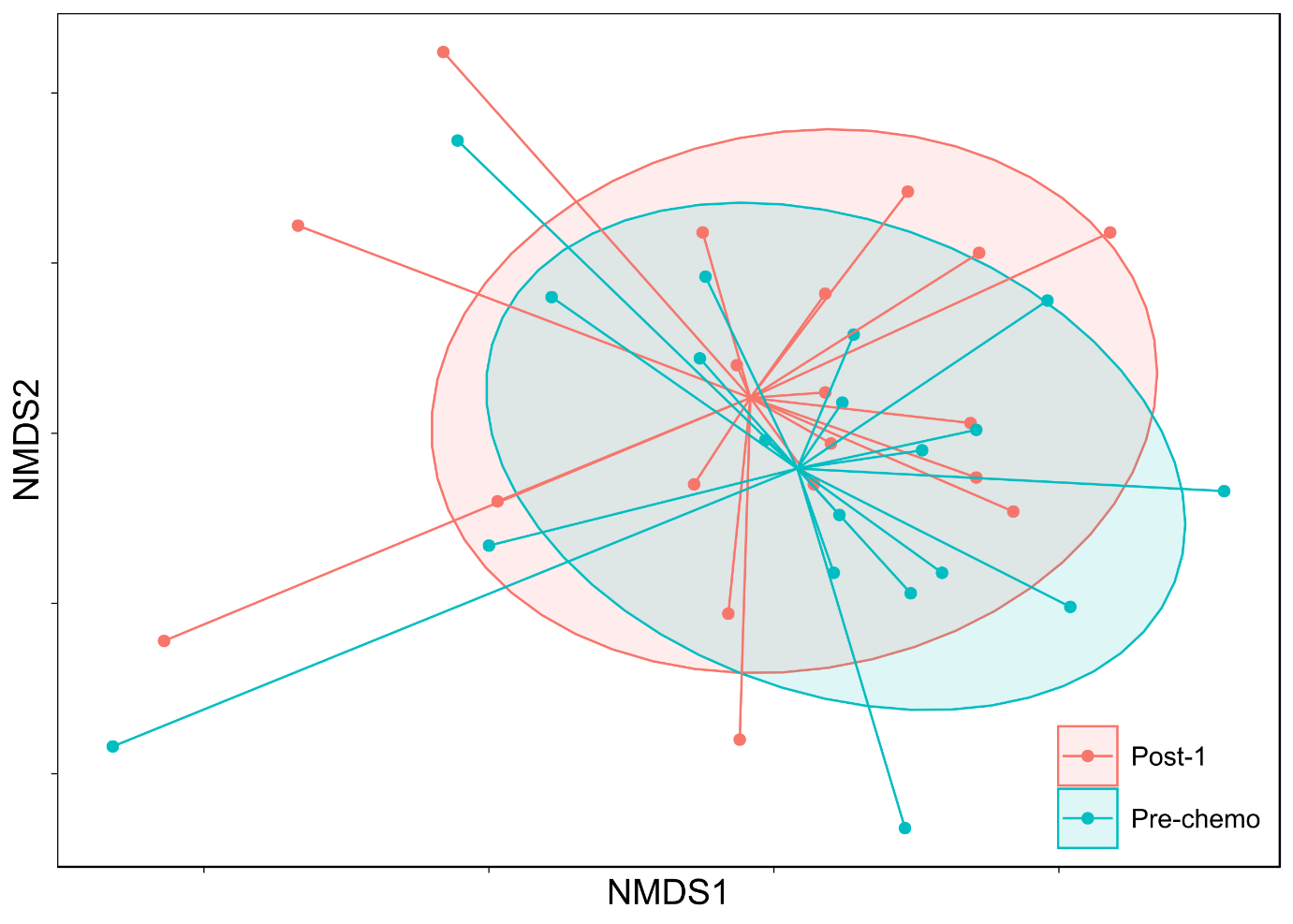


**Supplementary Figure 1.** Non-metric multi-dimensional scaling (nMDS) plot showing between group comparisons of faecal microbiome distribution and dispersal. nMDS plots depicted from Bray-Curtis resemblance of square root transformed, genus-level, relative abundance data. Shaded ovals represent 80% confidence interval. There is no difference between the pre-chemotherapy and post-chemotherapy (post-1) faecal microbiome distribution (PERMANOVA; p=0.99) or dispersion (PERMDISP; p=0.90).

**
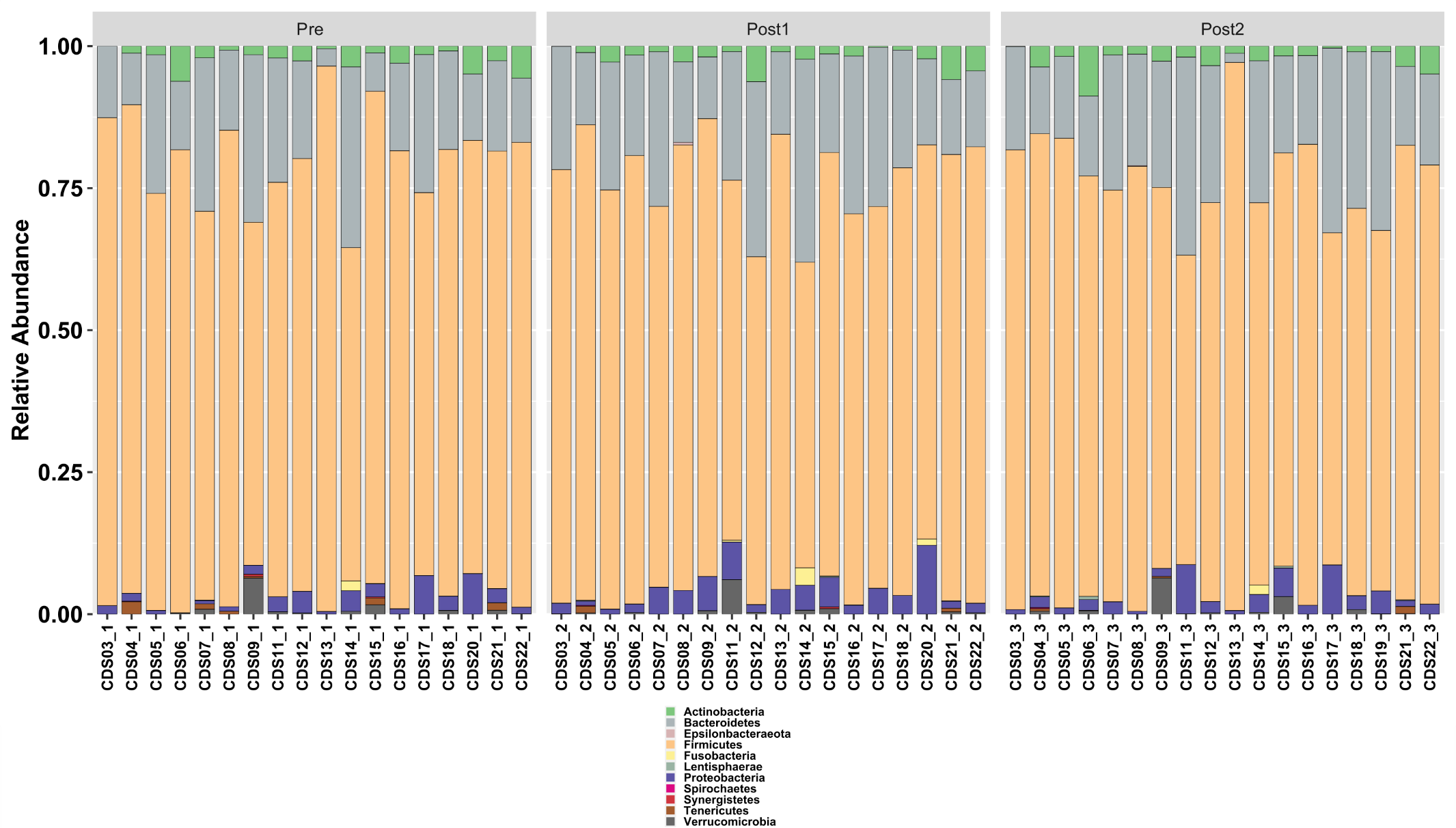
**

**Supplementary Figure 2.** Taxa bar plot showing the 11 phyla detected. Grouped by time point with Pre=pre-chemotherapy, Post1=7-12 days following chemotherapy, and Post2= at the end of a chemotherapy cycle (median 21 days post-chemotherapy). Showing predominance of Bacteroidetes and Firmicutes.

**
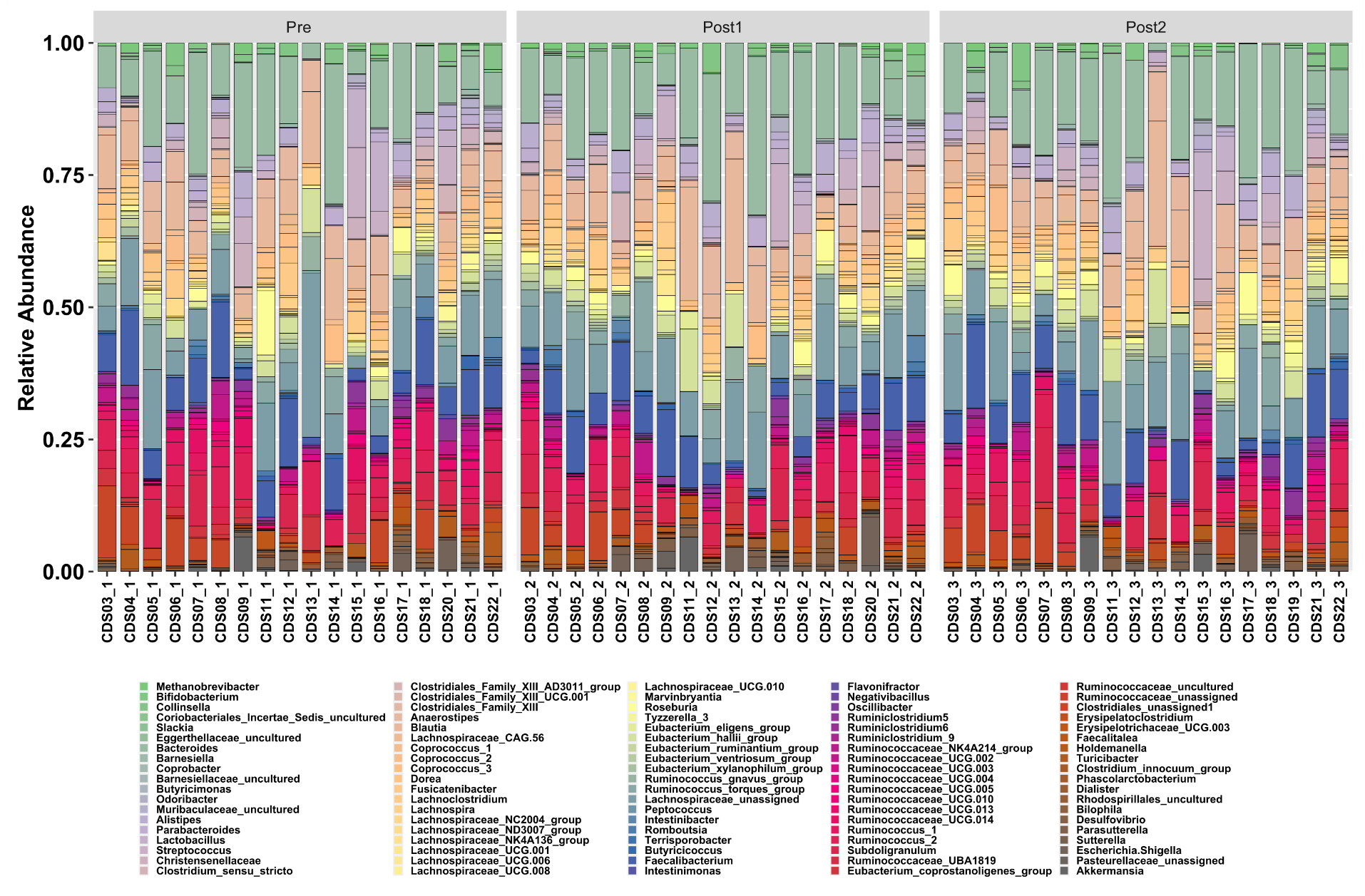
**

**Supplementary Figure 3.** Taxa bar plot showing the 95 genera detected in ≥20% of samples. Grouped by time point with Pre=pre-chemotherapy, Post1=7-12 days following chemotherapy, and Post2= at the end of a chemotherapy cycle (median 21 days post-chemotherapy).
